# Development of a Smartphone-Based System for Intrinsically Photosensitive Retinal Ganglion Cells Targeted Chromatic Pupillometry

**DOI:** 10.3390/bioengineering11030267

**Published:** 2024-03-09

**Authors:** Ana Isabel Sousa, Carlos Marques-Neves, Pedro Manuel Vieira

**Affiliations:** 1 Department of Physics, NOVA School of Science and Technology, NOVA University of Lisbon, 2829-516 Caparica, Portugal; pmv@fct.unl.pt; 2 Faculty of Medicine, University of Lisbon, 1649-028 Lisbon, Portugal; cmneves@fm.ul.pt

**Keywords:** pupil light reflex, pupillometry, intrinsically photosensitive retinal ganglion cells, smartphone, chromatic pupillometry

## Abstract

Chromatic Pupillometry, used to assess Pupil Light Reflex (PLR) to a coloured light stimulus, has regained interest since the discovery of melanopsin in the intrinsically photosensitive Retinal Ganglion Cells (ipRGCs). This technique has shown the potential to be used as a screening tool for neuro-ophthalmological diseases; however, most of the pupillometers available are expensive and not portable, making it harder for them to be used as a widespread screening tool. In this study, we developed a smartphone-based system for chromatic pupillometry that allows targeted stimulation of the ipRGCs. Using a smartphone, this system is portable and accessible and takes advantage of the location of the ipRGCs in the perifovea. The system incorporates a 3D-printed support for the smartphone and an illumination system. Preliminary tests were carried out on a single individual and then validated on eleven healthy individuals with two different LED intensities. The average Post-Illumination Pupil Light Response 6 s after the stimuli offsets (PIPR-6s) showed a difference between the blue and the red stimuli of 9.5% for both intensities, which aligns with the studies using full-field stimulators. The results validated this system for a targeted stimulation of the ipRGCs for chromatic pupillometry, with the potential to be a portable and accessible screening tool for neuro-ophthalmological diseases.

## 1. Introduction

The Pupil Light Reflex (PLR) to a light stimulus has been used for decades by clinicians to assess a person’s consciousness [1], either qualitatively using a swinging flashlight [2], or quantitatively through Automated Pupillometry [3,4,5]. Automated Pupillometry is the technique used to record and measure the pupil through time after a light stimulus.

The assessment of the pupil reactivity to light stimuli gained a renewed interest over the last two decades, especially with the discovery of melanopsin, a photopigment present in the intrinsically photosensitive Retinal Ganglion Cells (ipRGCs) [6,7]. These cells, through melanopsin, are sensitive to the absorption of the short-wavelength (blue) visible light [8], with a peak sensitivity at around 482 nm [8,9]. The ipRGCs were found to contribute to the PLR, combined with the other photoreceptors cones and rods [10]. Chromatic Pupillometry takes advantage of the sensitivities to different wavelengths in the visible light and measures the pupil response to blue and red stimuli. In healthy individuals, the PLR shows a sustained pupil recovery after the blue stimulus, in comparison to the red coloured stimulus [10], due to the ipRGCs activation. Chromatic Pupillometry has been shown relevant insights in the screening and detection of neuro-ophthalmological diseases [11,12,13,14], showing impaired PLR responses in patients with glaucoma and other optic neuropathies when compared to healthy individuals.

**Figure 1:**
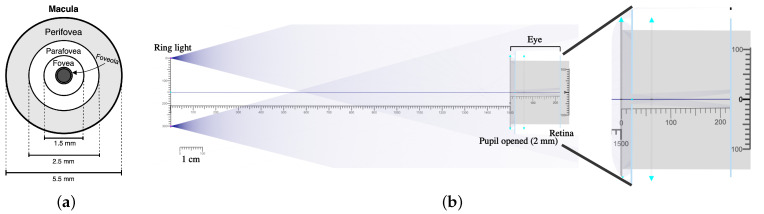
(**a**) Schema of the human macula. (**b**) Optic system simulation with 2 point sources distanced by 3 cm to simulate the ring light on the left and the eye model on the right side, with a distance of 15 cm between the ring light and the eye. On the right side of the figure, there is a zoomed image of the simulation showing that the light is entering the retina in the corresponding location of the perifovea.

The ipRGCs represent a small part of the retinal ganglion cells, around 0.4–1.5% [15]. The location of these cells, and consequently the melanopsin, is mainly in the perifoveal region where their concentration is higher, and they do not exist in the fovea [15]. The perifovea is the outer ring sliced area inside the macula, circumscribing the parafovea and fovea [16], as represented in the schema in Figure 1a. Standard commercial pupillometers that facilitate chromatic pupillometry recurrently use a full-field stimulator placed in front of the eye to trigger the coloured stimuli [10,17]. The full-field stimulators allow light to enter the retina at all angles; however, they are not taking advantage of the ipRGCs distribution in the retina, and the stimulus is not targeted. In a previous work [18], the authors proposed a targeted way to stimulate the ipRGCs, by using a ring-light properly configured and the right distance of the eye to target the perifovea, where ipRGCs are more abundant, using a traditional pupillometer. With the ring light stimulus, the study showed interesting results for chromatic pupillometry and was in agreement with the literature for healthy individuals.

The standard commercial pupillometers typically use near-infrared cameras and are expensive, not portable, less accessible and require the support of a trained operator. To explore the potential of using chromatic pupillometry as a widespread screening tool for neuro-ophthalmological diseases, these limitations need to be overcome. Smartphones, an accessible and easy-to-use technology, could have high interest as a solution to spread this technique and make it affordable and reachable. Some research has been presented over the last ten years for pupillometry using a smartphone [19,20,21,22]. In 2022, our group presented a preliminary study for chromatic pupillometry using a smartphone [21]. This work showed good-quality records of the eye and pupil detection with the smartphone. Although that system allowed blue and red stimuli using filters and the smartphone flashlight, the results did not show a pupil recovery after stimulus caused by the activation of the ipRGCs, with the blue stimulus, as observed in other studies [10,14,23] for healthy individuals. Using the flash light of the smartphone could be the issue in the previous work, as the light entering the retina was not targeted at the ipRGCs, which could be the reason for not seeing a consistent sustainable response of ipRGCs in the PLR with the blue stimulus.

Considering the idea of a ring-light for a targeted stimulation of the ipRGCs taking advantage of their higher concentration in the perifovea and the interest in having more portable and easy-to-use pupillometers, the present study proposes a new system for chromatic pupillometry that combines both. The aim was to develop a system that allows a targeted (ring-light) stimulation of the ipRGCs using a smartphone and its validation in healthy individuals.

## 2. Materials and Methods

### 2.1. Design and Development of the System

There are three main steps for the development of the system that will support targeted stimulation of ipRGCs in the retina and the usage of a smartphone camera for the acquisition: perform a simulation of the optic system to define the dimensions of the device and the support for the light stimuli, the actual design and 3D printing of the prototype and the electronics for the illumination system control.

#### 2.1.1. Optic System Simulation

Similarly to what was carried out in a previous work [18], an online open-source optic simulator named Ray Optics Simulation [24] was used for the optic system simulation. This simulation step was made to characterize and define the system total dimensions, especially to calculate the ring light size at a certain distance to the eye so the light illuminates the retina in the perifoveal region. In Figure 1b, there is a schema of the optical system simulation made for this study. Based on the location of the perifovea in the macula and its dimensions, as represented in Figure 1a, the goal was to have a ring light stimulus that would enter the retina in a radius between 1.25 and 2.75 mm from the center of the fovea [16]. The optical eye characteristics used in the simulation are the same as described in the previous work [18]. It was also considered for the simulation of a pupil size of 2mm, which is the minimum normal pupil size when there is a bright light [25]. The distance between the eye and the ring light was defined to be 15 cm, in a way that the smartphone camera can still focus and guarantee a sufficient quality of the images recorded. Then, an optimization was made using the simulator until finding the optimal ring light diameter that would allow a targeted stimulation of the ipRGCs. It was identified that the ring light needs to have around 3 cm of diameter to guarantee that the light enters in the perifoveal region.

Considering that the system would use the smartphone camera, the acquisitions cannot be made in total darkness, as the video recording would also be dark and no pupil could be detected. So an environment light is needed that would illuminate the eye but not interfere with the targeted stimulation of the ipRGCs. Therefore, the optic simulation was also used to assess if a tilted environment light placed bellow the eyesight would enter the eye in the perifoveal region and stimulating the ipRGCs.

#### 2.1.2. Design of the System and 3D Printing

A second part of the work was the design and 3D printing of the system that would support the smartphone and integrate the illumination system, in a way that the ring light is placed in front of the camera. The system was prepared to accommodate the smartphone Nokia 7 Plus (Nokia Corporation, HMD Global, Espoo, Finland) and using its rear-facing cameras for video recording. This smartphone model dimensions are 15.83 × 7.56 × 0.79, in centimeters. The support needs have two slots, one for the smartphone to fit and another to fit a piece in a circular format that will have the multi-colour LEDs (Light-Emitting Diodes), as the ring-light stimulus, and a support for the tilted environment light LEDs below. The ring-light stimulus support was designed to have 8 multi-coloured LEDs. The environment light should be achieved using 12 multi-coloured LEDs that would need a support for it. The LEDs were equally distributed in each illuminator support and their position allowed us to generate a uniformed light. Both pieces that would be part of the illumination system had to be prepared to receive the multi-coloured LEDs, with a 5 mm diameter. The LEDs used in this system are 5mm round Red and Blue and Pure Green with reference OSTAMC5B32A by OptoSupply, Hong Kong, China. According to the datasheet, these LEDs have a dominant wavelength of 470 ± 5 nm for the Blue LED and 625 ± 5 nm for the Red LED.

**Figure 2:**
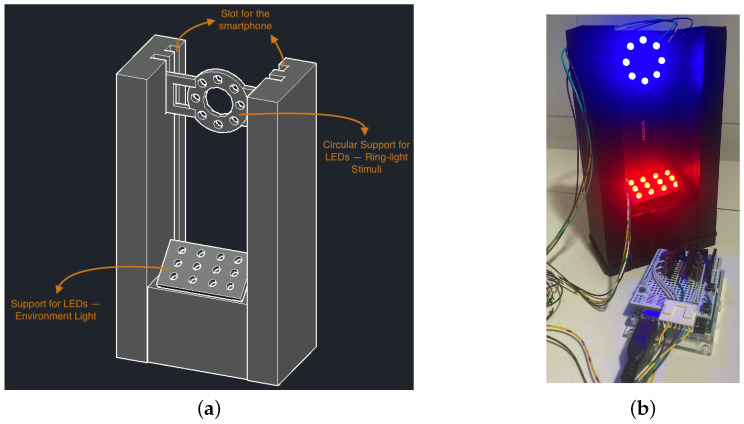
(**a**) Three-dimensional drawings of the support for the smartphone and the illumination system for the proposed system. (**b**) Developed system with 3D-printed support and the ring light and the environment light LEDs switched on.

In Figure 2a, there are the 3D drawings made using AutoCAD for Mac 2023 (version T.114.M.24, Autodesk) of the pieces and the full system prepared for 3D printing. All the dimensions have a margin of 0.75 mm to cover printing errors, especially in openings to fit the LEDs. The pieces of the support were then printed in a 3D printer (model CR-20 by Creality 3D) using a Polylactic Acid (PLA) filament, in black, so that it would reduce the reflection of any illumination sources.

#### 2.1.3. The Illumination System

The multi-coloured LEDs were connected in parallel in each of the illuminators (ring-light for the targeted stimulus and the environment light), so they could be controlled separately. Both illuminators were connected to an Arduino Uno Rev3 (Arduino, Somerville, MA, USA), and the connections were made using the 5V power of the Arduino. The illumination system is powered by a laptop, using the USB connection of the Arduino. Each colour of the LEDs (red, green and blue) was connected to a different output pin in Arduino, so the system could be prepared for all the possibilities in terms of light colour of the ring-light stimuli and the environment light. In Figure 2b, there is a picture of the illumination system connected to the Arduino.

This environment light was chosen to be red coloured, as per previous findings [21], with the main function of illuminating the individual’s eye and allowing good quality of the recorded image. A red coloured environment light, with a wavelength of 625 ± 5, is opposite to the ipRGCs peak sensitivity at around 482 nm [6,8,9] in the visible light spectrum, so it would not stimulate the ipRGCs neither impact their sustained pupillary response after stimulus.

A Graphical User Interface (GUI) was developed in Python language using the PySimpleGUI library (https://www.PySimpleGUI.com, accessed on 6 September 2021) to control the illumination system using the laptop. The GUI allows us to configure the illumination system according to the experiment and protocol that needs to be completed. Essentially, it allows us to switch ON and OFF both the ring light and the environment illuminator, set the ring-light stimuli duration and change the LEDs intensity.

### 2.2. Preliminary Tests and Adjustments to the Setup

The preliminary tests were made with one healthy individual (male, age 35 years old, iris colour: hazel) to confirm the distances of the eye and the quality of the video recorded using a red environment light. This was the same individual as the one from a previous work referred to in the conference paper [18], where promising results were obtained using a ring-light stimulus and a gold-standard pupillometer.

Different intensities of the ring-light LEDs were tested in these initial trials, for both blue and red colours: 6%, 14%, 22%, 29%, and 37% of the total intensity of the LEDs with this system. The LEDs datasheet information indicates that the maximum luminous intensity is 4200 ± 630 mcd for the red LED and 2000 ± 300 mcd for the blue LED, for the model used in this system. These experiments intended to understand what would be the illumination system optimal characteristics for the pupillometric measurements and ipRGCs targeted stimulation.

**Figure 3:**
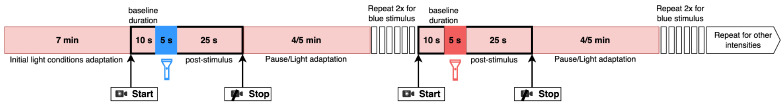
Acquisition protocol schema.

The protocol followed is based on previous works [18,20,21,26] and is represented in a schema in Figure 3. Initially, the individual was in a dark room for 7 min, with just the red environment light switched on. Then, the recording started using the smartphone camera, and after 10 s, the stimuli started with the LEDs in the ring light support switched on for 5 s. After the LEDs have been switched off, the recording continued for 25 s. Each experiment was repeated 3× for each stimulus/intensity. Between each measurement, there was a pause of 4/5 min to guarantee that the pupil had fully recovered. The red environment light was always on. The person focused on a target point placed in front at the same height as the smartphone, at a distance of around 1.5 m. The individual’s non-dominant eye was recorded, which was centred in front of the smartphone camera and the ring light. By recording the non-dominant eye, the individual could be comfortable focusing on the target, as the dominant eye would assume that responsibility, and the accommodation of the non-dominant eye was reduced.

### 2.3. Validation of the System in Healthy Individuals

Experiments were made in 11 healthy individuals (2 males, 9 females) with an age range between 18 and 32 years old, with an average age of 23.69 years old. In terms of eye colours of these of individuals, eight had brown irises, one had grey, one had green and another one had hazel. The acquisition protocol was the same as explained in the previous subsection, described in Figure 3.

To avoid photo-toxicity in all the individuals, we chose two intensities of the ring-light LEDs that showed interesting results in the preliminary tests made in one individual. These intensities were 14% and 37% of the total capacity of the LEDs. For each of these two intensities, acquisitions for the blue and red stimuli were made, repeated 3× for each.

### 2.4. Data Processing and Analysis

Data processing occurred after the acquisitions, using a computer. Each recorded video was analysed individually, and then, the results for each experimental scenario were averaged. For each video, all the frames were extracted, and in each frame, the pupil was detected, and its area was measured and saved.

In a single frame, after converting to grey scale, the eye region was cropped, and then, pupil detection algorithms were applied. For this, two algorithms were used: Pupil Reconstructor (PuRe), open-source and developed by Santini et al. [27], and a threshold-based algorithm developed by our research group. PuRe was applied in each frame, and in case it did not successfully detect a pupil, the threshold-based algorithm was applied. The full pupil detection process was described in a previous work [21]. The pupil response signal was then filtered, eye blinks and other outliers were removed by applying an exponential weighted moving average filter.

The PLR curves were based on the pupil area in pixels, normalized by the baseline, which is the average pupil size in the 10 s before the stimulus is switched on. The each value of the pupil size was applied to Equation (1), based on the Kelbsch et al. [23] equation for pupil area normalization.

(1) $$Normalized\;pupil\;area=100-\frac{baseline\;size-absolute\;size}{baseline\;size}\times 100$$

## 3. Results

**Figure 4:**
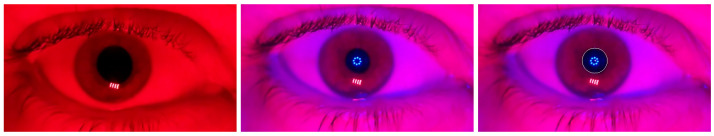
Samples of eye frames with the ring stimuli switched OFF, switched ON and with the pupil detected.

The videos acquired with the system developed in this work using the smartphone allowed applying pupil detection algorithms to each eye frame and collect the pupil area in each instance. The red environment light provides a good contrast between the iris and the pupil, even with different iris colours. In Figure 4, there are three samples of eye frames with the ring stimuli switched OFF, switched ON and with the pupil detected and drawn in the image, all with the red environment light switched on.

The preliminary tests carried out with one individual with five different intensities of the ring-light stimulus and the corresponding values of Post-Illumination Pupil Light Response 6 s after the stimulus offset (PIPR-6s) are represented in Table 1. There is a difference in the Post-Illumination PIPR-6s between the responses to red and blue stimuli in almost all the intensities, except in the highest (37% of the total intensity of the LEDs) and the 22%, as observed in the last column of Table 1.

**Figure 5:**
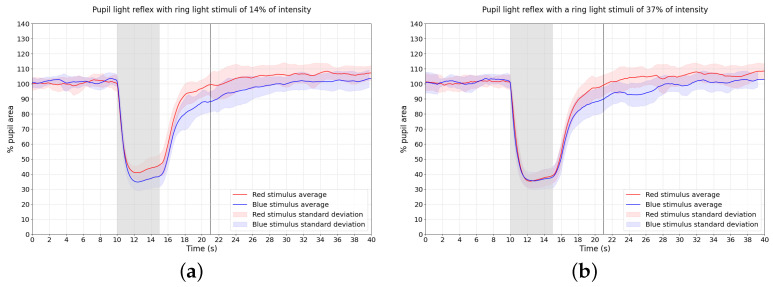
Average and standard deviation PLR curves for the 11 healthy individuals tested for the 2 intensities (**a**) 14% and (**b**) 37% for both blue and red colours. Gray area—period of time with stimuli on.

As for the validation in 11 healthy individuals, the average of the results are represented in two graphs in Figure 5 for red and blue stimuli at the two intensities tested: 14% and 37% of the total intensity of the LEDs. With the lowest intensity (6%), the individual from the preliminary tests had difficulties in keeping the focus on the target, and there was more variability in the pupil size during the experiments, which is the reason why this intensity was discarded for the validation study with healthy individuals. So, it was decided to use the 14% of the total intensity of the LEDs as well as the highest intensity of 37%, as an opposite. The PIPR-6s values for blue and red stimuli and the difference between them for each intensity is represented in Table 2.

## 4. Discussion

A sustainable recovery after the blue stimulus indicates the participation of the ipRGCs in the PLR, reflected in a difference between the blue and red recovery of the pupil after the stimulus [10,23]. Previous to this work, our group attempted to develop a system using only a smartphone for chromatic pupillometry, with special attention to the stimulation of the ipRGCs [21]. Although the systems proposed previously had visible pupillary responses to light stimuli, there was no sustained response after stimuli offset, a characteristic of the ipRGCs response to blue light. So, the developed smartphone-based system with a ring-light for targeted stimulation of the ipRGCs presented in this study could bring a novelty in this area of chromatic pupillometry and portable devices, as the results have shown indication of that.

Observing the pupil light response for blue and red stimuli of the 11 healthy individuals in Figure 5, the average curves show a visible difference in the pupil recovery after the stimulus between the blue and red stimuli for both the 14% and 37% intensities. This agrees with what was seen in the preliminary experiments with individual A. Both intensities showed PIPR-6s differences between red and blue stimuli concordant with what was seen in Park et al. [10]. In their work, a difference of around 11% is presented between the PIPR-6s for red and blue stimuli, for a stimulus with 10 s of duration. Furthermore, Feigl et al. [14] showed a difference in PIPR-6s of around 12% between red and blue stimuli in a healthy individual for a stimulus with 10 s as well. Similarly, a difference in the PIPR-6s of around 12% between red and blue stimuli was also shown in Kelbsch et al. [23] research for a stimulus with 4 s of duration and healthy individuals. Our results for the average of 11 individuals show a difference of 9.6% between red and blue PIPR-6s, as observed in Table 2, close values to what was observed in different research mentioned before where traditional pupillometers were used.

These are also concordant with the results obtained in preliminary experiments carried out using a traditional pupillometer and a ring-shaped stimulator on a conference paper presented by our research group [18]. In that previous work, a difference was obtained in the PIPR-6s of around 13.1% between red and blue stimuli. The interest and relevance of the system developed in the current research work in comparison to previous ones is the combination of using a smartphone as the main device for acquisition and supporting targeted stimulation of the ipRGCs. This brings portability, accessibility, and a reduction in costs of a system for ipRGCs targeted chromatic pupillometry.

During the stimulus onset, in the lower intensity tested (14%) in the 11 healthy individuals, it is observable that the maximum constriction for the blue light is higher than the red one, as observed in other works [10,14,17]. In the higher intensity, this difference in the PLR curves while the stimuli are on is not visible. In this case, the curves are almost the same from the beginning of the stimulus until approximately 1 s after the stimulus offset.

The proposed system implies using a red colour environment light to illuminate the individual’s eye during the measurements. Usually chromatic pupillometry is performed in the dark using infrared cameras. As we are obtaining similar results as seen using gold standard pupillometric systems, it can be an indicator that the red environment light does not have significant impact on the PLR sustained response caused by melanopsin in the ipRGCs for the blue stimulus.

It is relevant to mention that while performing the experiments, the individuals felt more comfort with the 14% intensity of the LEDs (the lower one tested). This should also be taken into consideration for the optimal characteristics of the protocol to be used further with the system proposed.

## 5. Conclusions

The validation made with the proposed system, using a ring-shaped stimulus, showed results concordant with the literature for chromatic pupillometry in healthy individuals. This ring-shaped approach to the light stimuli brings novelty as a way to stimulate the ipRGCs as it takes advantage of their higher concentration in the perifovea. It was also understood that the red environment light does not impact the pupillometric results and the activation of ipRGCs and helps provide good-quality video recording and eye image frames, allowing to use a common smartphone camera. Using the lower intensity of the LEDs that gives enough energy to stimulate the ipRGCs would be the most interest configuration for future work, as it gives expected results compared to the literature and it provides more comfort to the individuals. The developed system shows potential to be used for PLR with a targeted stimulation of ipRGCs using of a smartphone and a ring-shaped illuminator as stimuli generator. There is great interest in this novel approach to chromatic pupillometry for implementing this technique with a focus on ipRGCs stimulation and making it more accessible and useful for further studies.

As future work, it is intended to increase the number of healthy individuals in the measurements with the stimuli intensities used in this study for improved validity and reliability of the system in the control group. Although the results obtained were in agreement with what has been seen with other pupillometry systems, it would also be interesting to further study the illuminators design, especially the ring-shaped one, by testing different numbers of LEDs to assess what would be the optimal quantity of those to be distributed in the support. Further experiments to an optimization of the acquisition protocol would also be relevant for a steadier system, e.g., test different stimuli duration. This would be essential to the next step of the validation of the system in individuals with neuro-ophthalmologic diseases such as glaucoma. After validation in patients with neuro-ophthalmological diseases, this system has the potential to become an accessible and widespread screening tool for these types of pathologies.

## Figures and Tables

**Table 1:** PIPR-6s values for each intensity and stimulus colour in the preliminary tests for a single individual. Average of 3 measurements for each experiment.

Intensity	PIPR-6s Blue (%)	PIPR-6s Red (%)	Difference between Red and Blue (%)
6%	81.93 ± 12.96	92.72 ± 5.25	10.79
14%	80.16 ± 4.23	90.46 ± 11.1	10.30
22%	76.35 ± 0.98	77.26 ± 3.48	0.91
29%	73.98 ± 3.66	84.14 ± 2.71	10.16
37%	80.23 ± 8.00	85.32 ± 2.69	5.09

**Table 2:** PIPR-6s values for 14% and 37% intensities for the average of the 11 healthy individuals.

Intensity	PIPR-6s Blue (%)	PIPR-6s Red (%)	Difference between Red and Blue (%)
14%	88.15 ± 6.87	97.73 ± 7.56	9.58
37%	89.74 ± 7.97	99.13 ± 6.88	9.59

## Data Availability

The data presented in this study are available on request from the corresponding author.

## Abbreviations

The following abbreviations are used in this manuscript:

PLR	Pupil Light Reflex
ipRGCs	intrinsically photosensitive Retinal Ganglion Cells
LEDs	Light-Emitting Diodes
PIPR-6s	Pupil Response 6 s after the stimulus offset
